# Antisepsis for the Hands

**Published:** 1892-03

**Authors:** 


### Antisepsis for the Hands.

At the Johns Hopkins' Hospital the use of bichloride of mercury as an antiseptic has declined to a considerable extent, in favor of solutions of permanganate of potash in combination with oxalic acid. Dr. Malcom McLean, at the October meeting of the New York Obstetrical Society, reported on his use of three formulæ, given below, for obtaining an antiseptic condition of his hands (see *New York Journal of Gynecology* for December). Having briefly referred to the fact that Dr. W. H. Welch and other members of the Hopkins' surgical staff have come to the conclusion that corrosive sublimate solutions are inferior to those of the permanganate for many antiseptic purposes, the author says that he has found that the scrapings from the finger-nails, etc., taken after an ablution of the hands with any one of the ordinary antiseptic solutions, have developed, under culture in the laboratory, numerous germs. But when solutions of the permanganate of potash and oxalic acid had been used this was not the result, showing the superiority of the later agents. The staining of the hands by the potash solution has been a serious objection, but he believes that this may be obviated by the use of a solution of hyposulphite of soda, one part to sixteen, and oxalic acid, one part to thirty-two of water. The steps of Dr. McLean's process are (1), the hands, having been thoroughly cleaned, are to be held for two minutes in a solution of the permanganate of potash, five parts to one hundred, after which the hands should be rinsed in clear water; (2) hold the hands for one minute in a hypophosphite of soda solution, one ounce to the pint; and (3) while this is being done add the oxalic acid solution, one-half ounce to the pint of water. This causes a double chemical combination, whereby an oxalate of sodium and sulphur dioxide are formed, which have powerful decolorizing and disinfecting properties. The permanganate stains are promptly removed from skin and nails; after again rinsing the hands in sterilized water they are ready to come into contact with either an exposed serous or a lacerated mucous membrane. The hands may then be regarded as both surgically clean.

